# A227 VEDOLIZUMAB EXPOSURE ASSOCIATED WITH THE RESOLUTION OF OROFACIAL CROHN’S DISEASE: A CASE REPORT

**DOI:** 10.1093/jcag/gwae059.227

**Published:** 2025-02-10

**Authors:** K Alseiari, M Darling, M Mora, A Wilson

**Affiliations:** Gastroenterology, Western University Schulich School of Medicine & Dentistry, London, ON, Canada; Gastroenterology, Western University Schulich School of Medicine & Dentistry, London, ON, Canada; Gastroenterology, Western University Schulich School of Medicine & Dentistry, London, ON, Canada; Gastroenterology, Western University Schulich School of Medicine & Dentistry, London, ON, Canada

## Abstract

**Background:**

Orofacial Crohn’s disease (CD) is a rare and often debilitating manifestation of Crohn’s disease, characterized by deep ulcerations, cobblestone lesions of the buccal mucosa, and hallmark granulomas(1,6). Orofacial Crohn’s disease remains a challenging condition to manage, with limited available data on the use of advanced non-TNF-α therapies (3). Vedolizumab, a gut-selective integrin antagonist, has been shown to be effective for inflammatory bowel disease, but its role in treating orofacial CD has not been fully explored(2,3,5).

**Aims:**

The aim of this case report is to explore the potential efficacy of vedolizumab in treating orofacial Crohn’s disease, a rare and challenging manifestation of Crohn’s disease, by presenting a clinical case where vedolizumab led to significant resolution of both orofacial and gastrointestinal symptoms. This report aims to contribute to the limited data available on the use of non-TNF-α therapies, such as vedolizumab, in managing orofacial Crohn’s disease and to encourage further studies on its therapeutic role in this context.

**Methods:**

This is a case report of a 68-year-old female with a 33-year history of ileo-colonic Crohn’s disease developed severe orofacial CD after infliximab discontinuation due to peri-prosthetic infection. She presented with severe vomiting, oral ulcerations, and diarrhea, along with ileal inflammation and gastric outlet obstruction. Physical examination revealed angular stomatitis and cobblestone ulcers in the buccal mucosa and palate. Histopathology confirmed CD with granulomatous inflammation. Initial treatment with dexamethasone mouthwash and budesonide showed minimal improvement. The patient was transitioned to vedolizumab, receiving three induction doses followed by monthly maintenance.

**Results:**

Within two months of starting vedolizumab, the patient showed significant clinical and endoscopic improvement. By the fourth month, complete healing of the oral ulcerations was achieved, alongside resolution of her gastrointestinal symptoms, including gastric outlet obstruction. Vedolizumab was well-tolerated with no significant adverse effects reported.

**Conclusions:**

This case demonstrates the potential efficacy of vedolizumab in treating orofacial Crohn’s disease, a rare and challenging presentation of Crohn’s disease. Nonetheless, vedolizumab binds to the α4β7 integrin, blocking its interaction with MAdCAM-1 on the surface of gastrointestinal endothelial cells (5). Since MAdCAM-1 is also expressed in the oral mucosa (4), we believe that vedolizumab may offer a promising alternative for patients with orofacial Crohn’s disease who do not respond to traditional therapies. Further studies are needed to establish its role in managing this condition

**Funding Agencies:**

None

